# Supplementary material for: Draft genome sequence of the pulse crop blackgram [Vigna mungo (L.) Hepper] reveals potential R-genes
Source: Sci Rep. 2021 May 27;11:11247. doi: 10.1038/s41598-021-90683-9 (PMC8160138; doi:10.1038/s41598-021-90683-9)
Supplement: Supplementary file 1 — Supplementary Table S1. [file 41598_2021_90683_MOESM1_ESM.docx]

**Draft genome sequence of the pulse crop blackgram [*Vigna mungo* (L.) Hepper] reveals potential R-genes.**

Souframanien J ^12^, Avi Raizada^2^, Punniyamoorthy Dhanasekar^1^ and Penna Suprasanna^1^

^1^ Nuclear Agriculture and Biotechnology Division, BARC, Trombay, Mumbai-400085, India.

^2^Homi Bhabha National Institute, Training School Complex, Anushaktinagar, Mumbai-400094, India.

**Table S1:** Raw data statistics of blackgram genome reads generated by Illumina HiSeq and ONT.

| **Sample** | **Platform** | **Library and chemistry** | **No. of raw reads** | **No. of processed reads** | **Coverage** |
| --- | --- | --- | --- | --- | --- |
| SO_8668_PE | HiSeq | PE (150 x 2) | 154940012 | 140116780 | 98x |
| SO_8668_MP_5-7 kb | HiSeq | MP (150 x 2) | 33617232 | 26742270 | 21x |
| SO_8668_MP_7-10 kb | HiSeq | MP (150 x 2) | 10247813 | 7586306 | 6.5x |
| SO_8668_NP | ONT | Long read | 1633898 | 1633786 | 22x |

Abbreviations: kb, kilobases; PE, paired-end; MP, mate-pair; ONT, Oxford Nanopore Technology.
